# Dysregulated monocyte compartment in PACS patients

**DOI:** 10.3389/fimmu.2025.1613034

**Published:** 2025-06-06

**Authors:** Romy Kronstein-Wiedemann, Madeleine Teichert, Elisa Michel, Janina Berg, George Robinson, Kristin Tausche, Martin Kolditz, Johannes Bergleiter, Jessica Thiel, Dirk Koschel, Stephan R. Künzel, Kristina Hölig, Torsten Tonn, Manuela Rossol

**Affiliations:** ^1^ Laboratory for Experimental Transfusion Medicine, Transfusion Medicine, Faculty of Medicine Carl Gustav Carus, Technische Universität Dresden, Dresden, Germany; ^2^ Institute for Transfusion Medicine, German Red Cross Blood Donation Service North-East, Dresden, Germany; ^3^ Molecular Immunology, Faculty of Health Sciences, Brandenburgische Technische Universität (BTU) Cottbus-Senftenberg, Senftenberg, Germany; ^4^ Division of Pneumology, Medical Department I, University Hospital Carl Gustav Carus and Technische Universität (TU) Dresden, Dresden, Germany; ^5^ East German Lung Center/Ostdeutsches Lungenzentrum, Dresden-Coswig, Germany; ^6^ Department of Transfusion Medicine, Medical Clinic and Polyclinic I, University Hospital Carl Gustav Carus, Dresden, Germany; ^7^ Department of Internal Medicine and Pneumology, Fachkrankenhaus Coswig, Lung Center, Coswig, Germany; ^8^ Institute for Transfusion Medicine and Immunohematology, Goethe University Hospital Medical School, German Red Cross Blood Donor Service, Frankfurt, Germany; ^9^ Faculty of Environment and Natural Sciences, Brandenburgische Technische Universität (BTU) Cottbus-Senftenberg, Senftenberg, Germany

**Keywords:** monocytes, intermediate monocytes, CD56+ monocytes, COVID-19, PACS, SARS-CoV-2

## Abstract

**Introduction:**

1-5% of all patients with COVID-19, a disease caused by infection with Severe Acute Respiratory Syndrome Virus 2 (SARS-Cov-2), even those with mild COVID-19 symptoms, continue to have symptoms after initial recovery. Symptoms associated with the post-acute sequelae of COVID-19 (PACS) include, among others, fatigue, shortness of breath, cough, and cognitive dysfunction. Since the dysregulated immune response appears to be caused by the sustained activation of certain immune cells, including monocytes, and the release of specific cytokines, the aim of our study was to investigate the effect of PACS disease on monocyte subpopulations.

**Methods:**

Twenty-two healthy and thirty-two patients with PACS were included into this study. We performed blood gas analysis and measured hematological parameters from peripheral blood of PACS patients and compared them with healthy donors. Surface markers to identify monocyte subpopulations were analyzed by flow cytometry.

**Results:**

PACS patients had higher numbers of intermediate and CD56+ monocytes, whereas the numbers of total monocytes, classical and non-classical monocytes were normal compared to healthy donors. Comparison of patients with and without fatigue, cough, and dyspnea showed no difference in monocyte subset frequencies. However, patients with cognitive dysfunction had increased numbers of non-classical monocytes compared to patients without this symptom.

**Discussion:**

This suggests a disturbed homeostasis of the monocyte subsets in the peripheral blood of patients with PACS.

## Introduction

1-5% of all people infected with severe acute respiratory syndrome coronavirus 2 (SARS-CoV-2) develop long-lasting symptoms, collectively referred to as post-acute sequelae of SARS-CoV-2 infection (PACS) or Long-COVID (1–3). Symptoms include fatigue, cognitive dysfunction, and pulmonary complications, such as shortness of breath (dyspnea) and chronic cough (4). In addition, PACS patients show a persistent low grade inflammation (5–8). Overall, the symptoms are similar to other post-infectious conditions (9, 10). As patients with PACS vary considerably in terms of symptoms, severity and recovery profile, attempts have been made to distinguish different clinical phenotypes, but no consensus has yet been reached (11, 12) and the underlying cause of PACS symptoms has remained a mystery.

Human monocytes can be divided into three major populations: classical (CD14^++^CD16^-^), intermediate (CD14^++^CD16^+^), and non-classical (CD14^+^CD16^+^) monocytes (13), of which classical monocytes in particular express high levels of the ACE-2 receptor (14), which is one of the cellular entry receptor for SARS-CoV-2 (15). Monocytes and macrophages are involved in all phases of the response to viral infections (16–18). Infection with SARS-CoV-2 leads to an altered monocyte phenotype in the acute phase (19–23), and the alterations can predict the severity of the disease (24, 25). Monocytes are also implicated in the pathogenesis of PACS. PACS patients have elevated monocyte-platelet aggregates (7), dysregulated monocyte subpopulations (5, 26–28), show signs of activation (8, 28), and might be a reservoir for persistent infection (27).

In this study, we analyzed peripheral blood monocyte subpopulations in PACS patients. We found increased absolute numbers of intermediate monocytes and CD56+ monocytes. In addition, non-classical monocytes were increased in PACS patients with cognitive dysfunction and decreased in PACS patients without cognitive dysfunction compared to healthy controls. Moreover, D-dimer concentrations were higher in PACS patients with cognitive dysfunction than in patients without this symptom. The monocyte compartment alterations in PACS patients suggest a dysregulation of monocyte fate which might contribute to the persistence of Long-COVID symptoms.

## Materials and methods

### Patients and controls

Blood samples from 32 PACS patients were provided by the Post-COVID center of the Department of Pulmonology, Medical Department I (University Hospital Carl Gustav Carus, Dresden, Germany). The patients were referred because they showed persisting symptoms after SRAS-CoV-2 infection, and the diagnosis of PACS was made according to the German national guideline for Long-COVID and included documentation of previous SARS-CoV-2 infection, standardized symptom and functional assessment, and standardized exclusion of other causes (29). All patients suffered previously from mild COVID-19 infection according to World Health Organization (WHO) criteria for the clinical management of COVID-19. Fatigue was assessed using the Fatigue Severity Scale (FSS) and the Fatigue Assessment Scale (FAS). The FSS has been shown to demonstrate high internal consistency, validity and sensitivity to changing clinical conditions (30). The FAS questionnaire consists of 10 items answered on a 5-point Likert scale ranging from 1 (never) to 5 (always) (31). Sleepiness was measured using the Epworth Sleepiness Score (ESS). The ESS is a widely used, validated questionnaire and has been shown to be a reliable measure of persistent daytime sleepiness in adults (32). The PACS symptom cognitive dysfunction was self-reported by the patients and was included in the analysis with present or not present.

Age- and sex-matched blood samples from healthy donors (n=22) were provided by the German Red Cross Blood Donation Service North-East, Institute for Transfusion Medicine Dresden and by the Department of Pulmonology, Medical Department I (University Hospital Carl Gustav Carus, Dresden, Germany).

### Blood collection and sampling

Six ml whole blood from healthy donors and from Long-COVID patients were collected in S-Monovettes EDTA K3 (Sarstedt, Nümbrecht, Germany) by venipuncture using a sterile disposable Safety-Multifly-Needle 21 G (Sarstedt). Blood cell numbers were determined using a Sysmex XN 1000 (Sysmex Deutschland GmbH, Norderstedt, Germany). The normal reference range for monocyte numbers is 200–1000 monocytes per µl blood. Measurement of COHb was carried out as part of the blood gas analyzing with an ABL800 Flex (Radiometer Medical ApS, Brønshø, Denmark). D-dimer was measured using the STA R Max^®^ (Stago, Düsseldorf, Germany) and blood was collected in citrate monovettes (Sarstedt).

### Ethics approval

Blood samples from all donors and Long-COVID patients were used in anonymized form and in accordance with the guidelines approved by the Ethics Committee of the Technical University of Dresden [BO-EK-49012022]. Informed consent was obtained from all donors and patients.

### Flow cytometry

50µL whole blood was incubated with PE-labeled anti-CD14 (clone MφP9, BD Bioscience, Heidelberg, Germany), APC-labeled anti-CD56 (clone NCAM16.2, BD Bioscience) and BV421-labeled anti-CD16 (clone 3G8, BD Bioscience) for 15 minutes at room temperature. Samples were then incubated in 450µL BD FACS™ Lysing Solution for 15 min, vortexed and analyzed by flow cytometry using a FACSLyric flow cytometer (BD Bioscience) with FlowJo software. The gating strategy to identify the monocyte subsets within all CD14+ monocytes has been described previously (33). Absolute numbers of monocyte subsets were calculated using the absolute monocyte count of the blood.

### Graphs and statistics

Graphs and statistics were prepared with GraphPad Prism 10.2.2. Bar charts represent mean + s.e.m. and individual values of each experiment are represented as symbols in bars. Statistical significance was determined accordingly using the two-tailed non-parametric, unpaired Mann-Whitney U tests, confidence interval of 95%. For multiple comparisons, the Kruskall-Wallis test with Dunn’s test was performed and adjusted p-values were used.

## Results

The clinical characteristics of patients with post-acute sequelae of COVID-19 (PACS) and healthy donors are presented in **Table 1**.

**Table 1:** Clinical characteristics of the study participants.

Parameters	Healthy controls	PACS
No. (Sex, F/M)^1^	22 (11/11)	32 (23/9)
Age, y^2^	48.5 ± 2.7	47.0 ± 2.2
Age, range	27-69	29-79
BMI, kg/m^2^	ND	26.9 ± 4.9
Onset (days)	NA	565.3 ± 385
CRP (mg/L)	ND	3.2 ± 4.1
Chronic fatigue	NA	23 (71.9%)
Dyspnea	NA	26 (81.3%)
Cough	NA	23 (71.9%)
Cognitive dysfunction	NA	9 (28.1%)
FSS	NA	51.7 ± 10.6
FAS^*^	NA	33.6 ± 7.2
ESS^<ns/>^	NA	12.2 ± 4.4

All values are expressed as mean ± SEM. ND not determined, NA not applicable, ^*^ seven data points missing, ^<ns/>^ one data point missing, ^1^ no significant difference between healthy controls and PACS patients, Chi-square test, ^2^ no significant difference between healthy controls and PACS patients, Mann-Whitney test.

**Figure 1:**
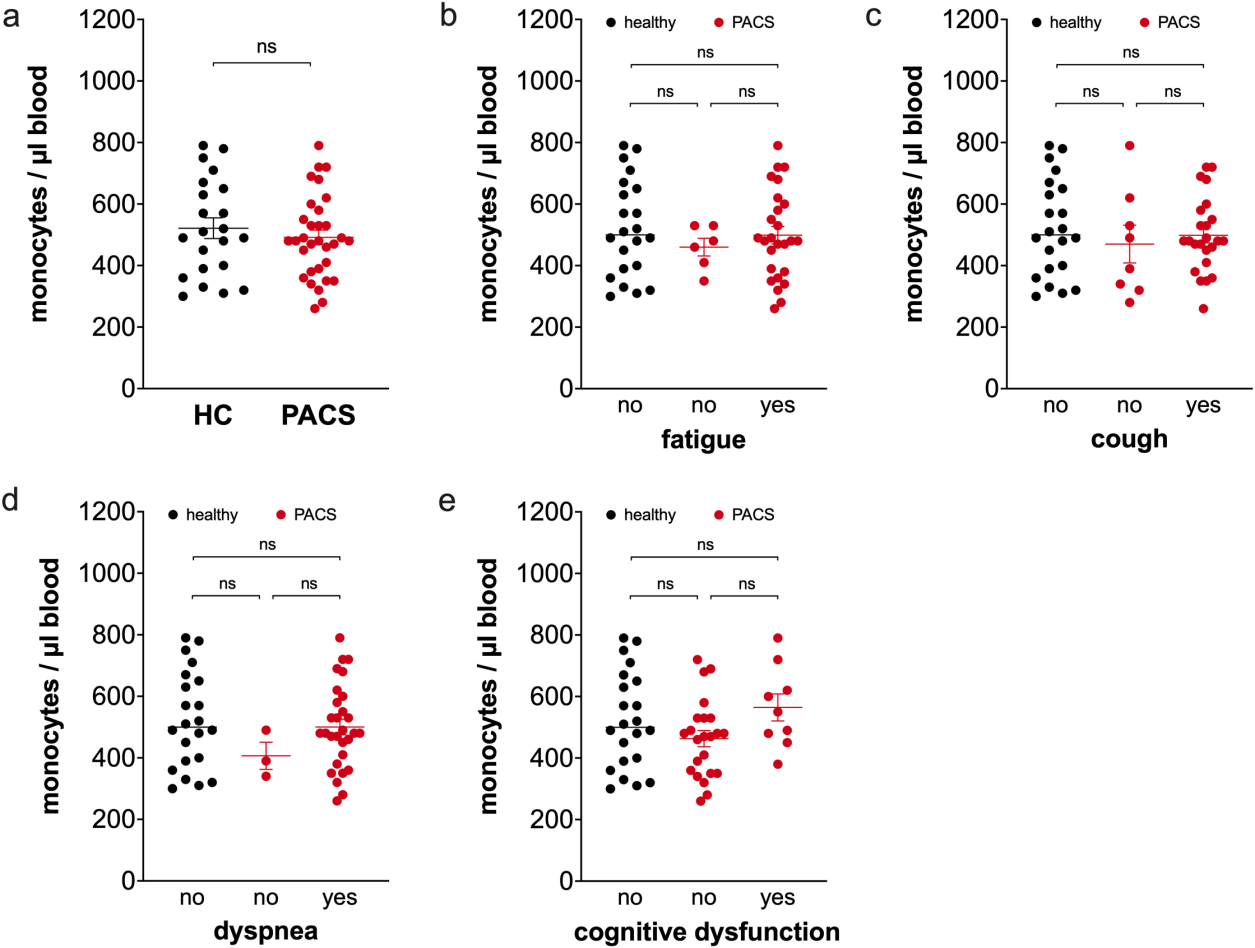
Comparison of total monocyte numbers of healthy controls and PACS patients. **(a)** Absolute numbers of total monocytes of healthy controls (HC, *n* = 22) and PACS patients (PACS, *n* = 32). Scatter plots show mean ± SEM. **(b)** Absolute numbers of total monocytes of healthy controls (HC, *n* = 22) and PACS patients without fatigue (*n* = 6) and with fatigue (*n* = 26). **(c)** Absolute numbers of total monocytes of healthy controls (HC, *n* = 22) and PACS patients without cough (*n* = 8) and with cough (*n* = 24). **(d)** Absolute numbers of total monocytes of healthy controls (HC, *n* = 22) and PACS patients without dyspnea (*n* = 3) and with dyspnea (*n* = 29). **(e)** Absolute numbers of total monocytes of healthy controls (HC, *n* = 22) and PACS patients without cognitive dysfunction (*n* = 23) and with cognitive dysfunction (*n* = 9). Scatter plots show mean ± SEM. Statistical analysis was performed using two-tailed Mann–Whitney *U* test **(a)** or Kruskall-Wallis test with Dunn’s test **(b-e)**. ns, not significant.

Absolute monocyte numbers in the peripheral blood were indistinguishable between healthy controls and PACS patients (**Figure 1a**). Comparisons of patients with and without fatigue (**Figure 1b**), cough (**Figure 1c**), dyspnea (**Figure 1d**), and cognitive dysfunction (**Figure 1e**) revealed that total monocytes were not different between the two groups.

**Figure 2:**
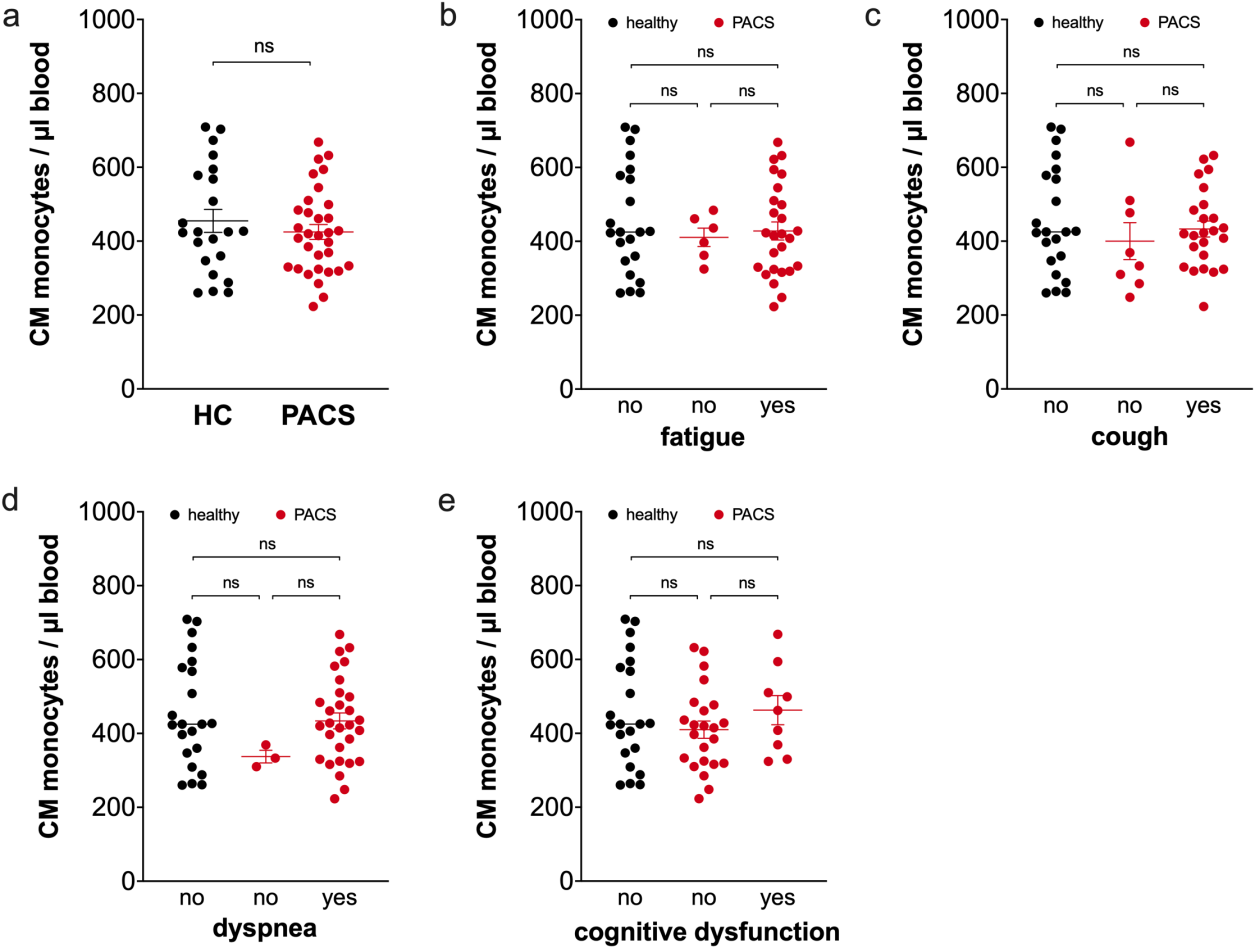
Comparison of classical monocyte numbers of healthy controls and PACS patients. **(a)** Absolute numbers of classical monocytes of healthy controls (HC, *n* = 22) and PACS patients (PACS, *n* = 32). Scatter plots show mean ± SEM. **(b)** Absolute numbers of classical monocytes of healthy controls (HC, *n* = 22) and PACS patients without fatigue (*n* = 6) and with fatigue (*n* = 26). **(c)** Absolute numbers of classical monocytes of healthy controls (HC, *n* = 22) and PACS patients without cough (*n* = 8) and with cough (*n* = 24). **(d)** Absolute numbers of classical monocytes of healthy controls (HC, *n* = 22) and PACS patients without dyspnea (*n* = 3) and with dyspnea (*n* = 29). **(e)** Absolute numbers of classical monocytes of healthy controls (HC, *n* = 22) and PACS patients without cognitive dysfunction (*n* = 23) and with cognitive dysfunction (*n* = 9). Scatter plots show mean ± SEM. Statistical analysis was performed using two-tailed Mann–Whitney *U* test **(a)** or Kruskall-Wallis test with Dunn’s test **(b-e)**. ns, not significant.

PACS patients and healthy controls had equal percentages of classical monocytes (86.6% ± 0.9 vs. 86.9% ± 0.9, ns) in total monocytes. Calculations of absolute numbers of classical monocytes also showed no difference between PACS patients and controls (**Figure 2a**). PACS patients with fatigue (**Figure 2b**), with cough (**Figure 2c**), with dyspnea (**Figure 2d**), or with cognitive dysfunction (**Figure 2e**) had the same classical monocyte numbers than patients without the respective symptoms.

**Figure 3:**
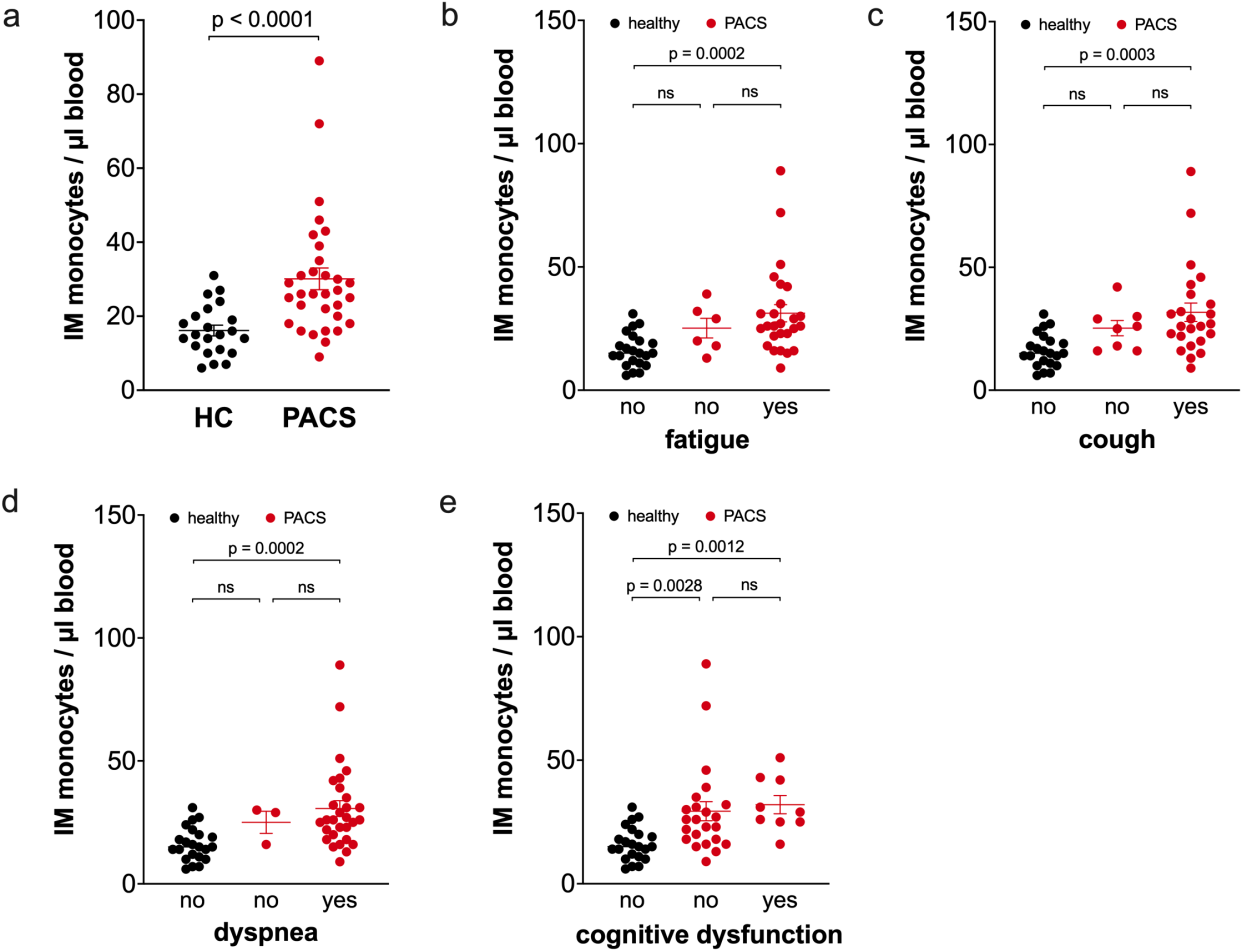
Comparison of intermediate monocyte numbers of healthy controls and PACS patients. **(a)** Absolute numbers of intermediate monocytes of healthy controls (HC, *n* = 22) and PACS patients (PACS, *n* = 32). Scatter plots show mean ± SEM. **(b)** Absolute numbers of intermediate monocytes of healthy controls (HC, *n* = 22) and PACS patients without fatigue (*n* = 6) and with fatigue (*n* = 26). **(c)** Absolute numbers of intermediate monocytes of healthy controls (HC, *n* = 22) and PACS patients without cough (*n* = 8) and with cough (*n* = 24). **(d)** Absolute numbers of intermediate monocytes of healthy controls (HC, *n* = 22) and PACS patients without dyspnea (*n* = 3) and with dyspnea (*n* = 29). **(e)** Absolute numbers of intermediate monocytes of healthy controls (HC, *n* = 22) and PACS patients without cognitive dysfunction (*n* = 23) and with cognitive dysfunction (*n* = 9). Scatter plots show mean ± SEM. Statistical analysis was performed using two-tailed Mann–Whitney *U* test **(a)** or Kruskall-Wallis test with Dunn’s test **(b-e)**. ns, not significant.

PACS patients had a higher percentage of intermediate monocytes in total monocytes than healthy controls (6.0% ± 0.4 vs. 3.2% ± 0.3, p<0.0001), and as shown in **Figure 3a** the absolute number of intermediate monocytes was also increased. This expansion of intermediate monocytes is independent of PACS symptoms fatigue (**Figure 3b**), cough (**Figure 3c**), dyspnea (**Figure 3d**), and cognitive dysfunction (**Figure 3e**). We observed no difference in the number of intermediate monocytes between PACS patients with a disease duration >2 years and <2 years (median 26 cells/µl blood, n=19 vs. median 26 cells/µl blood, n=13, ns).

**Figure 4:**
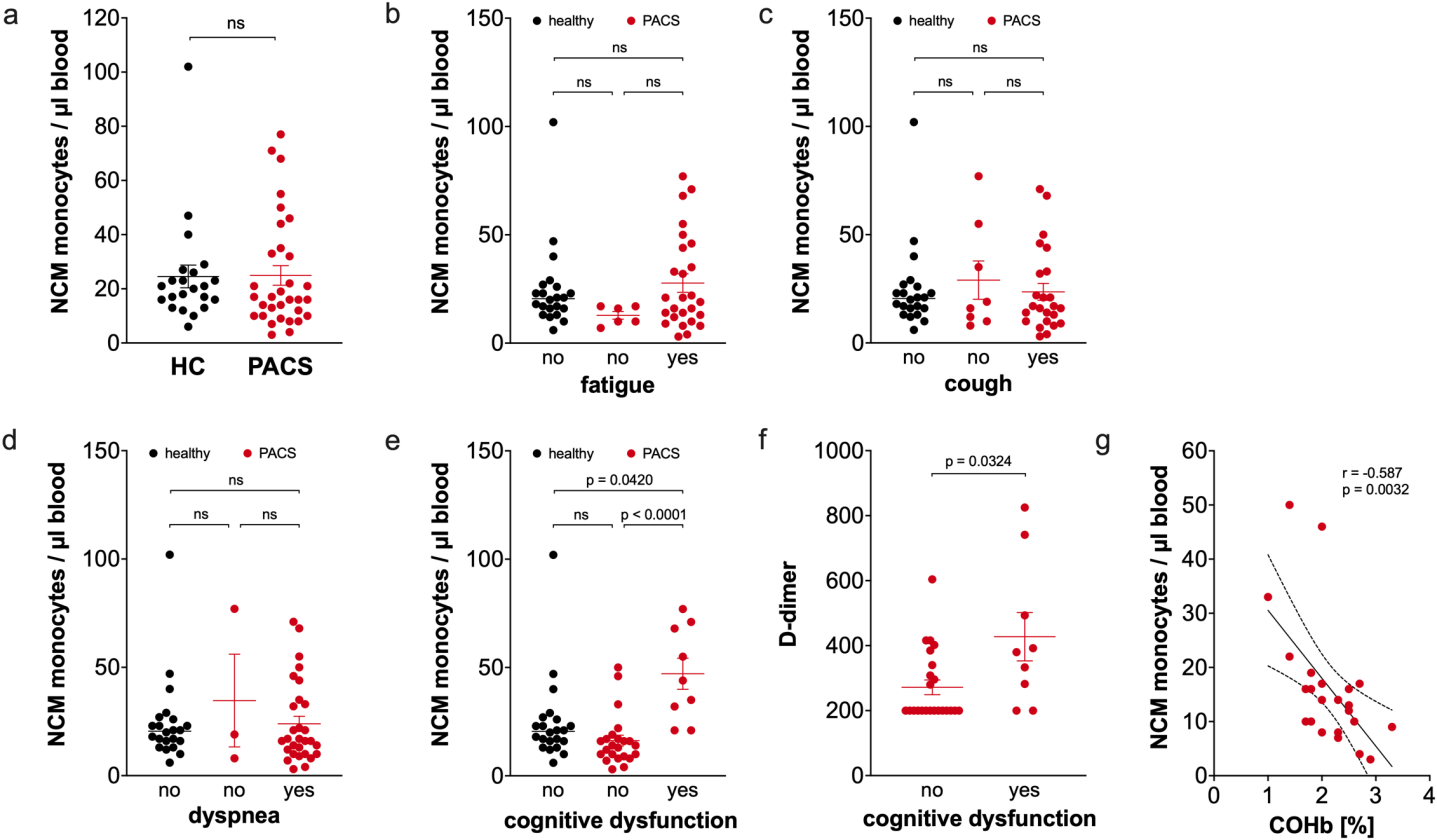
Comparison of non-classical monocyte numbers of healthy controls and PACS patients. **(a)** Absolute numbers of non-classical monocytes (NCM) of healthy controls (HC, *n* = 22) and PACS patients (PACS, *n* = 32). Scatter plots show mean ± SEM. **(b)** Absolute numbers of non-classical monocytes of healthy controls (HC, *n* = 22) and PACS patients without fatigue (*n* = 6) and with fatigue (*n* = 26). **(c)** Absolute numbers of non-classical monocytes of healthy controls (HC, *n* = 22) and PACS patients without cough (*n* = 8) and with cough (*n* = 24). **(d)** Absolute numbers of non-classical monocytes of healthy controls (HC, *n* = 22) and PACS patients without dyspnea (*n* = 3) and with dyspnea (*n* = 29). **(e)** Absolute numbers of non-classical monocytes of healthy controls (HC, *n* = 22) and PACS patients without cognitive dysfunction (*n* = 23) and with cognitive dysfunction (*n* = 9). **(f)** D-dimer concentration in the serum of PACS patients without cognitive dysfunction (*n* = 23) and with cognitive dysfunction (*n* = 9). **(a-f)** Scatter plots show mean ± SEM. Statistical analysis was performed using two-tailed Mann–Whitney *U* test **(a, f)** or Kruskall-Wallis test with Dunn’s test (b-e). **(g)** Correlation of absolute numbers of non-classical monocytes of PACS patients without cognitive dysfunction with carboxylated hemoglobin (COHb) (*n* = 23). Spearman correlation coefficient and level of significance as indicated. ns, not significant.

The percentage of non-classical monocytes in total monocytes was not different between PACS patients and healthy controls (5.0% ± 0.7 vs. 4.9 ± 0.8, ns). The absolute numbers of non-classical monocytes also did not differ between PACS patients and healthy controls (**Figure 4a**). We also observed equal non-classical monocyte numbers in PACS patients with and without fatigue (**Figure 4b**), cough (**Figure 4c**), and dyspnea (**Figure 4d**). However, we observed a striking difference between PACS patients with cognitive dysfunction and without (**Figure 4e**). PACS patients with cognitive dysfunction had more non-classical monocytes than PACS patients without cognitive dysfunction and healthy controls.

It has been previously reported that D-dimer levels in acute COVID-19 predict the development if cognitive dysfunction in PACS patients (34). We analyzed D-dimer levels in our cohort and found increased concentrations in the blood of PACS patients with cognitive dysfunction in comparison to patients without (**Figure 4f**). However, only two patients with cognitive dysfunction and one patient without cognitive dysfunction had clinical relevant D-dimer concentrations above the cutoff of 500 µg/ml. Carbon monoxide binding to hemoglobin, previously described by us to be elevated in PACS patients (35), negatively correlated strongly with non-classical monocyte numbers in PACS patients without cognitive dysfunction (**Figure 4g**), whereas no correlation was observed in PACS patients with cognitive dysfunction (**Supplementary Figure 1**).

**Figure 5:**
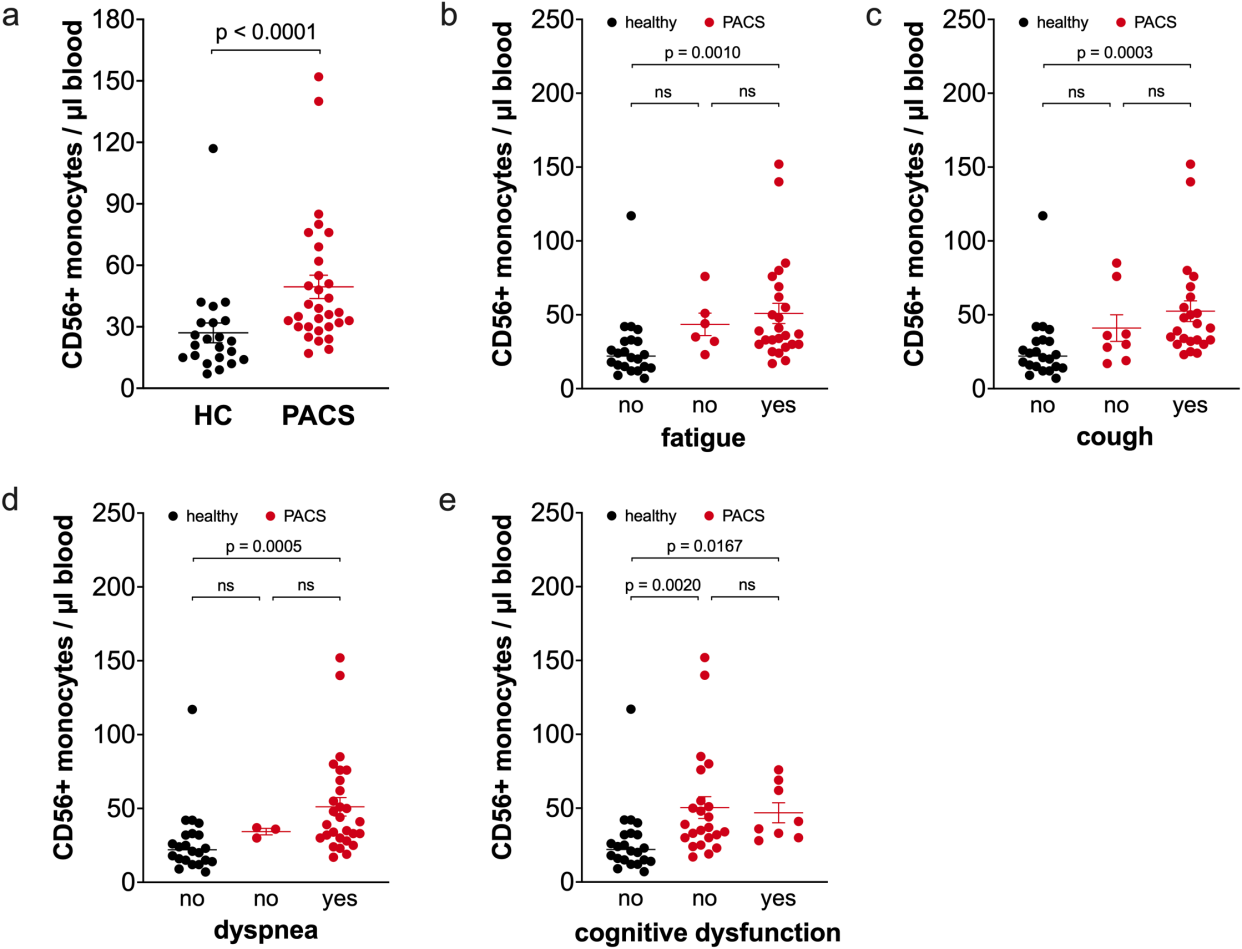
Comparison of CD56+ monocyte numbers of healthy controls and PACS patients. **(a)** Absolute numbers of CD56+ monocytes of healthy controls (HC, *n* = 22) and PACS patients (PACS, *n* = 32). Scatter plots show mean ± SEM. **(b)** Absolute numbers of CD56+ monocytes of healthy controls (HC, *n* = 22) and PACS patients without fatigue (*n* = 6) and with fatigue (*n* = 26). **(c)** Absolute numbers of CD56+ monocytes of healthy controls (HC, *n* = 22) and PACS patients without cough (*n* = 8) and with cough (*n* = 24). **(d)** Absolute numbers of CD56+ monocytes of healthy controls (HC, *n* = 22) and PACS patients without dyspnea (*n* = 3) and with dyspnea (*n* = 29). **(e)** Absolute numbers of CD56+ monocytes of healthy controls (HC, *n* = 22) and PACS patients without cognitive dysfunction (*n* = 23) and with cognitive dysfunction (*n* = 9). Scatter plots show mean ± SEM. Statistical analysis was performed using two-tailed Mann–Whitney *U* test **(a)** or Kruskall-Wallis test with Dunn’s test **(b-e)**. ns, not significant.

We also included the CD56+ monocyte subpopulation in our analysis. This subpopulation is part of the classical monocyte subpopulation and expands during aging, obesity, and in autoimmune diseases (33, 36, 37). PACS patients had a higher percentage of CD56+ monocytes (10.5% ± 1.4 vs. 5.2% ± 0.7, p<0.0001) in total monocytes than healthy controls. Calculations of absolute numbers of CD56+ monocytes revealed that PACS patients had more CD56+ monocytes than healthy controls (**Figure 5a**). We reported previously that the CD56+ monocyte subpopulation expands with age (36). We observed this again in the healthy controls (**Supplementary Figure 2a**), but there was no correlation of CD56+ monocytes and age within the PACS cohort (**Supplementary Figure 2b**). The CD56+ monocytes were already expanded in young PACS patients compared to age-matched controls (median 34 cells/µl blood, n=11 vs. 16 cell/µl blood, n=7, p = 0.0121, age <40 years). The number of CD56+ monocytes was equal between PACS patients with a disease duration >2 years and <2 years (median 37 cells/µl blood, n=19 vs. median 38 cells/µl blood, n=12, ns). The increase in CD56+ monocyte numbers is independent of PACS symptoms fatigue (**Figure 5b**), cough (**Figure 5c**), dyspnea (**Figure 5d**), and cognitive dysfunction (**Figure 5e**).

## Discussion

Our findings indicate that intermediate monocytes and CD56+ monocytes are expanded in PACS patients. These monocyte compartment perturbations were also found in PACS patients with a disease duration greater than two years, suggesting a persistent and long-term dysregulation of the monocyte subsets. Peripheral blood monocyte subpopulations circulate in a dynamic equilibrium (38). Classical monocytes emerge from the bone marrow and circulate for one day. 99% of classical monocytes leave the circulation, the other 1% then transition to intermediate monocytes which circulate for four days. Finally, all intermediate monocytes mature to non-classical monocytes which circulate for seven days. This results in approximately 87-92% classical monocytes, 3-5% intermediate monocytes, and 5-8% non-classical monocytes in the peripheral blood monocyte pool. In a state of inflammation, the differentiation from classical monocytes into intermediate and non-classical monocytes can occur over a shorter timespan (38).

Increased numbers of intermediate monocytes in PACS patients have been reported previously. Park et al. reported about increased total monocyte numbers and increased numbers of all three monocyte subpopulations (28), whereas Patterson et al. observed an increased percentage of intermediate monocytes (27). The increase in intermediate monocytes in our PACS cohort was not associated with any specific PACS symptom but was present in all PACS patients. Intermediate monocytes are found in increased proportions during acute Sars-CoV-2 (20–22) infection, other viral infections (39, 40), and many inflammatory diseases like rheumatoid arthritis (41), obesity (33), and sepsis (42). We among others have reported that intermediate monocytes are the main producers of pro-inflammatory cytokines (41, 43). In addition, intermediate monocytes are the main producers of reactive oxygen species (44), they express high levels of HLA-DR, CD80, and CD86 (41, 45), indicating their role in antigen presentation, and CCR5 (41, 43, 45), a chemokine receptor.

With the dynamic equilibrium of the monocyte subsets in the peripheral blood and the subsequent differentiation of all intermediate monocytes into non-classical monocytes (38), we expected to see also an increased proportion of non-classical monocytes. This was only true for PACS patients with cognitive dysfunction, all other patients showed normal non-classical monocyte numbers. While other studies observed increased percentages or numbers of non-classical monocytes in PACS patients (26, 27), it is interesting to note that COVID-19 patients show a depletion of non-classical monocytes in the peripheral blood as well as in the airways (19, 20, 22, 23, 46). Previously, we reported that PACS patients had impaired oxygen-hemoglobin binding and enhanced carbon monoxide binding (35). In the current study, non-classical monocyte numbers in PACS patients without cognitive dysfunction negatively correlated with carboxyhemoglobin (COHb). COHb is also increased in COVID-19 (47), respiratory infections (48), and sepsis (49), and endogenously derived from the metabolism of heme by heme oxygenase.

We observed a striking increase in non-classical monocyte numbers in PACS patients with a cognitive dysfunction. However, it is important to note, as a limitation of our study, that the cognitive dysfunction was a self-reported symptom and no additional tests were performed. In general, there is no objective cognitive marker for PACS. Recently, cognitive slowing was described by Zhao et al. as such a marker that is easy to evaluate using two short web-based cognitive tasks (50). A systematic study on non-classical monocytes and cognitive dysfunction in a larger cohort of PACS patients needs to be done to gain more insight into these findings.

Non-classical monocytes in general are known to be involved in the innate surveillance of tissues and in the response to viruses (43). The murine counterpart of human non-classical monocytes was found to be patrolling the endothelium of blood vessels and be involved in the resolution of inflammation (43, 51). The non-classical monocyte subset has been implicated to play a role in the cognitive impairment of aging people with HIV (52) and in the steady-state immune surveillance of the central nervous system (53). In COVID-19, some patients develop neurological sequelae and those patients have monocytes in the cerebrospinal fluid (54), and PACS patients with cognitive dysfunction also show recruitment of monocytes into the cerebrospinal fluid (55).

D-dimer is a fibrin degradation product and we found increased D-dimer concentrations in PACS patients with cognitive dysfunction in comparison to PACS patients without this symptom. The presence of D-dimers are associated with a cognitive decline in older people (56, 57), and Taquet et al. linked elevated D-dimer relative to C-reactive protein in acute COVID-19 to subjective cognitive deficits and occupational impact in PACS patients (34). There is some evidence for thromboinflammatory dysregulation in PACS patients. Increased D-dimer concentrations have been reported in PACS patients (58–61) as well as coagulation factor 11 (7). PACS patients have circulating fibrinolysis-resistant microclots (62), platelet-monocyte aggregates (7), and persistent complement dysregulation (7).

The combination of unchanged total monocyte numbers, the increase in intermediate monocytes and normal non-classical monocyte numbers in the majority of patients suggest a disturbed homeostasis of the monocyte subsets in the peripheral blood or an increased extravasation of non-classical monocytes in PACS patients. Cervia-Hasler et al. reported about the down-regulation of the transcription factor NR4A1 in classical monocytes of PACS patients (7). NR4A1 is a transcription factor that is necessary for the maturation of non-classical monocytes (63) and a down-regulation could point to a disturbed homeostasis of the monocyte subsets. There is also some evidence for an increased recruitment of monocytes into tissues in PACS patients. Scott et al. describe a monocyte migration profile in PACS patients that would promote migration of monocytes into the lung (64). They found an increased expression of the chemokine receptor CXCR6 on monocytes of PACS patients with unresolved lung injury and the ligand CXCL16 is abundantly expressed in the lung. Cheong et al. showed that monocytes and hematopoietic stem and progenitor cells (HSPC) showed epigenomic reprogramming up until one year following severe COVID-19 (65). The monocytes had a more pronounced pro-inflammatory response, and they found an increased recruitment of monocytes into the lung and brain in a mouse model. The focus of this study was not on PACS patients, however, Cheong et al. re-analyzed data from post-mortem lung tissue from post-acute COVID-19 patients from a study by Rendeiro et al. (66), and found an increased accumulation of monocytes in the lung. Interestingly, Rendeiro et al. observed a persistent presence of SARS-CoV-2 epitopes in the lung of the patients up to 359 days after the acute phase.

We also observed a marked increase of CD56+ monocytes in patients with PACS. The increase of CD56+ monocytes was found in all PACS patients except patients without cough. CD56+ monocytes, a subpopulation within classical monocytes, are expanded during healthy aging (36), in autoimmune diseases such as rheumatoid arthritis (36) and Crohn’s disease (37), in obesity (33), and in cancer (67). They produce more reactive oxygen intermediates and pro-inflammatory cytokines, and are more efficient antigen-presenting cells (36, 37, 68). CD56+ monocytes have also been described in acute COVID-19. Campana et al. observed an increased frequency of CD56+ monocytes in intensive care unit (ICU) patients but not in non-ICU COVID-19 patients (46). They also described them as hyperinflammatory, because the CD56+ monocytes produced more pro-inflammatory cytokines. Dutt et al. also reported an increase of CD56+ monocytes in COVID-19 patients (69). There is not much known about the CD56+ monocyte subset in the convalescence phase. Ravkov et al. observed no difference between convalescent COVID-19 patients and healthy controls, however, they only included convalescent patients who had a mild form of the disease (70).

We have described previously that the CD56+ monocyte subset expands with age (36). We could replicate this finding again in the healthy controls in this study. However, the CD56+ monocyte subset in PACS patients did not show any dependence on age, and more importantly the expansion of the subset was also found in young PACS patients. This could point to a premature aging of the monocytes (71), we observed this also in patients with rheumatoid arthritis (36). Lo Tartaro et al. reported about an increase in CD56+ monocytes in aged patients (>70 years old) with severe COVID-19 pneumonia in comparison to younger patients (<60 years old) and healthy controls (72). However, they also observed increased CD56+ monocyte numbers in the younger patients compared to healthy controls, suggesting that the expansion of the subset is also not age-dependent in acute COVID-19.

The appearance of CD56+ monocytes during aging is most likely caused by the low-grade inflammation, and we have reported about the expanded CD56+ subset in obese patients, another low-grade inflammatory disease (33). A low-grade inflammation has also been described in PACS patients (5, 7). In addition to the effect of low-grade inflammation, the highly inflammatory events during acute COVID-19 can have long-lasting effects to lead to epigenetic re-programming of the monocytes (65, 73).

In conclusion, this study shows that PACS patients have increased numbers of intermediate monocytes and CD56+ monocytes, while non-classical monocyte numbers were found to be increased in PACS patients with cognitive dysfunction. This suggests a disturbed homeostasis of the monocyte subsets in the peripheral blood of patients with PACS.

## Data Availability

The raw data supporting the conclusions of this article will be made available by the authors, without undue reservation.
