# Supplementary material for: Dysregulated monocyte compartment in PACS patients
Source: Front Immunol. 2025 Jun 6;16:1613034. doi: 10.3389/fimmu.2025.1613034 (PMC12179141; doi:10.3389/fimmu.2025.1613034)
Supplement: Supplementary file 1 [file DataSheet1.pdf]

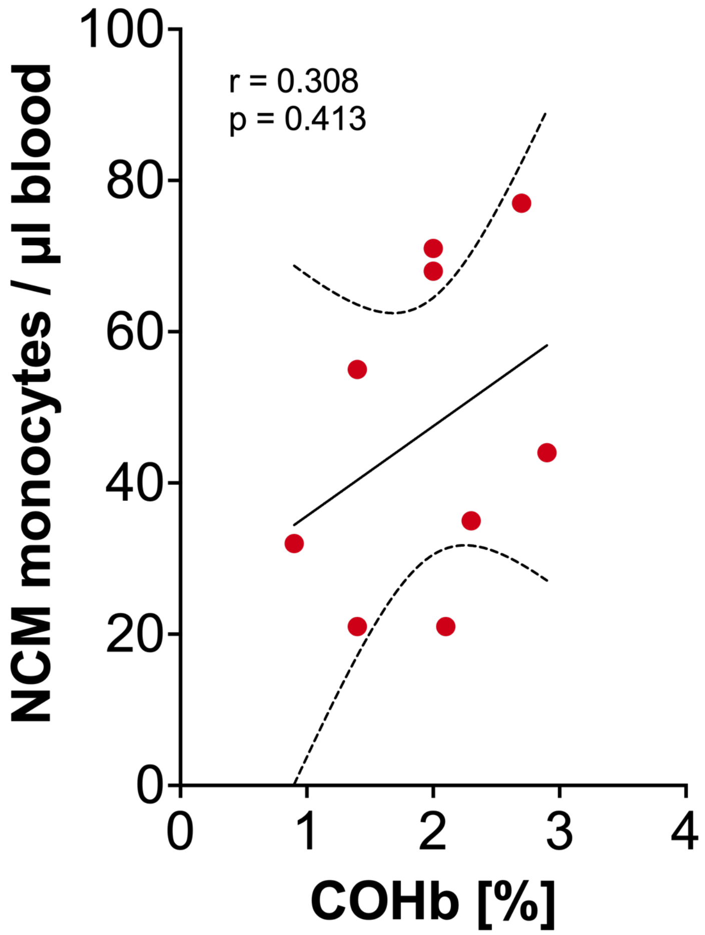

Supplemental Figure 1: Correlation of absolute numbers of non-classical monocytes (NCM) of PACS patients with cognitive dysfunction with carboxylated hemoglobin (COHb) (n = 8). Spearman correlation coefficient and level of significance as indicated.

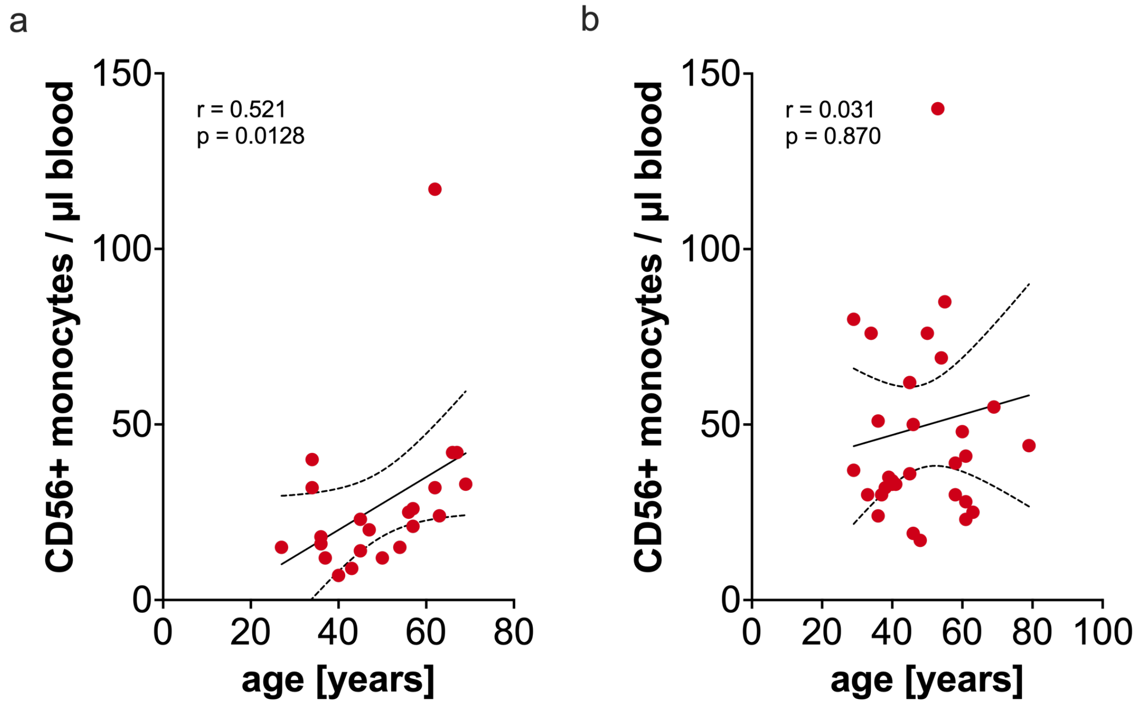

Supplemental Figure 2: Correlation of absolute numbers of CD56+ monocytes of healthy controls (a) and PACS patients (b) with age. Spearman correlation coefficient and level of significance as indicated.
